# Telomeric Repeat-Containing RNAs (TERRA) Decrease in Squamous Cell Carcinoma of the Head and Neck Is Associated with Worsened Clinical Outcome

**DOI:** 10.3390/ijms19010274

**Published:** 2018-01-17

**Authors:** Valerio Vitelli, Paolo Falvo, Solomon G. Nergadze, Marco Santagostino, Lela Khoriauli, Paola Pellanda, Giulia Bertino, Antonio Occhini, Marco Benazzo, Patrizia Morbini, Marco Paulli, Camillo Porta, Elena Giulotto

**Affiliations:** 1 Department of Biology and Biotechnology, University of Pavia, 27100 Pavia, Italy; valerio.vitelli@ifom.eu (V.V.); Paolo.Falvo@ieo.it (P.F.); solomon.nergadze@unipv.it (S.G.N.); marco.santagostino@unipv.it (M.S.); lela.khoriauli@unipv.it (L.K.); pellanda.paola@gmail.com (P.P.); 2 Department of Otorhinolaryngology, IRCCS San Matteo University Hospital Foundation, 27100 Pavia, Italy; g.bertino@smatteo.pv.it (G.B.); a.occhini@smatteo.pv.it (A.O.); m.benazzo@smatteo.pv.it (M.B.); 3 Department of Molecular Medicine, University of Pavia, 27100 Pavia, Italy; patrizia.morbini@unipv.it (P.M.); marco.paulli@unipv.it (M.P.); 4 Division of Medical Oncology, IRCCS San Matteo University Hospital Foundation, 27100 Pavia, Italy; c.porta@smatteo.pv.it

**Keywords:** telomere transcription, TERRA, head and neck squamous cell carcinoma

## Abstract

Telomeres are transcribed into noncoding telomeric repeat-containing RNAs (TERRA), which are essential for telomere maintenance. Deregulation of TERRA transcription impairs telomere metabolism and a role in tumorigenesis has been proposed. Head and neck cancer (HNC) is one of the most frequent cancers worldwide, with head and neck squamous cell carcinoma (HNSCC) being the predominant type. Since HNSCC patients are characterized by altered telomere maintenance, a dysfunction in telomere transcription can be hypothesized. In this prospective study, we compared TERRA levels in the tumor and matched normal tissue from 23 HNSCC patients. We then classified patients in two categories according to the level of TERRA expression in the tumor compared to the normal tissue: (1) lower expression in the tumor, (2) higher or similar expression in tumor. A significant proportion of patients in the first group died of the disease within less than 34 months postsurgery, while the majority of patients in the second group were alive and disease-free. Our results highlight a striking correlation between TERRA expression and tumor aggressiveness in HNSCC suggesting that TERRA levels may be proposed as a novel molecular prognostic marker for HNSCC.

## 1. Introduction

Telomeres are nucleoprotein structures located at the ends of eukaryotic chromosomes. Telomeres are essential for the maintenance of genome stability, preventing chromosome ends from being recognized as double-strand breaks and assuring the proper replication of chromosomes. In vertebrates, telomeric DNA consists of extended arrays of the hexamer TTAGGG. Telomeric DNA is bound by a specific multiprotein complex called Shelterin, which ensures proper regulation and protection of telomeres [1]. Telomeres are transcribed into long, noncoding telomeric repeat-containing RNAs (TERRA) [2,3,4]. A large body of evidence supports the hypothesis that TERRA plays a fundamental role in telomere maintenance and deregulation of TERRA synthesis causes genomic instability and telomeric defects [2,5,6,7,8,9,10,11,12], therefore, the study of TERRA expression in pathologies, in which telomere metabolism is impaired, is particularly relevant. In an early study, the analysis of TERRA levels in three different human tumor samples (from larynx, colon and B cell lymphoma) suggested that telomeric transcription is downregulated in advanced tumors [3]. A similar result was obtained by Sampl and collaborators [13] in human astrocytomas while, in mouse medulloblastoma, TERRA was overexpressed compared to normal tissues [14].

Head and neck cancer (HNC) is a frequent type of cancer, with more than 500,000 new cases per year being diagnosed wordwide [15]. In spite of the fact that combined and aggressive therapies are currently used, including pre- and postoperative chemotherapy, radiotherapy, and more recently also immunotherapy, there has been no significant improvement in five-year survival over the past 20 years. Treatment failures occur as locoregional recurrence, distant metastasis, and/or second primary tumors [16,17]. HNCs include several types of cancer originating from the site, the predominant type consists of squamous cell carcinomas (95%), while 4.5% are salivary gland adenocarcinomas or melanomas [18]. Within the HNC family, the head and neck squamous cell carcinoma (HNSCC) is a particularly aggressive malignant tumor type arising from the epithelial lining of mucosal membranes of the upper-aerodigestive tract (i.e., pharynx, hypopharynx and larynx) and the oral cavity. HNSCC is a clinically, pathologically, phenotypically and biologically heterogeneous disease deriving from multiple cellular and molecular alterations of the squamous epithelium. The main and well-established risk factors for HNSCC are smoking and alcohol abuse; these habits jointly multiply the risk of cancer, especially in the mouth and pharynx.

A growing body of evidence pinpoints the involvement of tumor-suppressor genes related the regulation of DNA replication and cell growth, including E2F, p16 and p53, in the progression of HNSCC. Notably, several studies suggested that approximately half of HNSCC tumors contain p53 mutations [19,20,21]. In addition, it was shown that human papilloma virus (HPV) is involved in the pathogenesis of a subgroup of HNSCCs. HPV is a risk factor for cancer of the oropharynx, particularly in tumors that are not associated with p53 mutations or with other risk factor such as tobacco and alcohol [22,23]. Based on this evidence it has been suggested that oropharyngeal cancer develops through two different pathways: the first one related to tobacco/alcohol consumption and p53 mutation, and the second one to HPV infection and genome instability.

A wide heterogeneity in terms of clinical outcome and response to treatment has been described. Thus, new prognostic and predictive markers are under investigation [24], including microRNAs [25,26].

In HNSCC patients, both cancer cells and cells from the mucosa surrounding the tumor are characterized by telomere shortening and genomic instability [27,28,29], supporting the hypothesis that telomere dysfunction drives genomic instability during the early events in the HNSCC oncogenic process. High levels of telomerase expression are found in 75–100% of HNSCCs [29,30,31,32,33], with the level of activity increasing together with the tumor stage [28]. To our knowledge, no information is available on telomeric transcription status in HNSCC.

In this prospective study, we compared TERRA levels in the tumor of 23 HNSCC patients with those of the adjacent normal tissue and found a correlation between telomere transcription levels and tumor aggressiveness.

## 2. Results

In this prospective study we analyzed TERRA levels in 23 patients affected by HNSCC. For all patients, we extracted total RNA from tumor tissue and from adjacent normal tissue. For three patients, RNA was extracted also from metastatic tissue of laterocervical lymphnodes. Total TERRA levels were measured by slot blot, using a (CCCTAA)_5_ oligonucleotide as probe [2,4,34]. For each patient, TERRA level in the tumor was compared to the value from the adjacent normal tissue.

**Figure 1:**
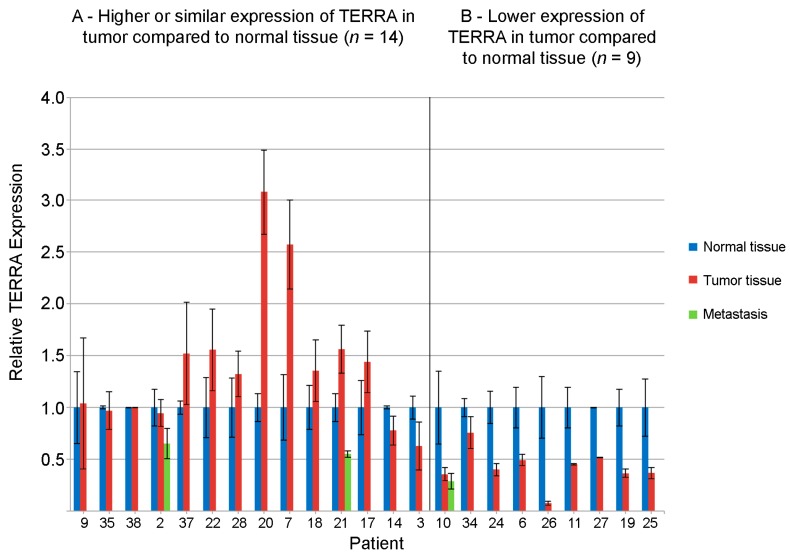
Quantification of total Telomeric Repeat-Containing RNA (TERRA) levels by slot blot hybridization in 23 head and neck squamous cell carcinoma (HNSCC) patients. Tumor samples (red bars), samples from metastatic tissue (green bars) and samples from adjacent nontumoral tissue (blue bars). For each patient, the value of the normal tissue has been set to 1. In group (**A**), the value in the tumor sample was either similar to the value in the healthy tissue (*p* > 0.05) (patients 9, 35, 38, 2, 37, 28, 14, 3) or was significantly higher (*p* < 0.05) (patients 22, 20, 7, 18, 21, 17). In group (**B**), the value in the tumor was significantly lower (*p* < 0.05) than in the healthy tissue.

The results are reported in Figure 1. In fourteen patients, TERRA expression values in the tumor were higher or similar to those of the adjacent normal tissue, while in nine patients TERRA expression was lower in the tumor sample. In the three metastatic samples, TERRA levels were always lower than in the normal tissue independently on the expression in the primary tumor. It is important to point out that, as described in the Methods section, assignment of patients n. 14, 3 and 34 to the two classes should be taken with caution because their *t*-test *p*-values were close to the 0.05 significance cut-off value.

**Figure 2:**
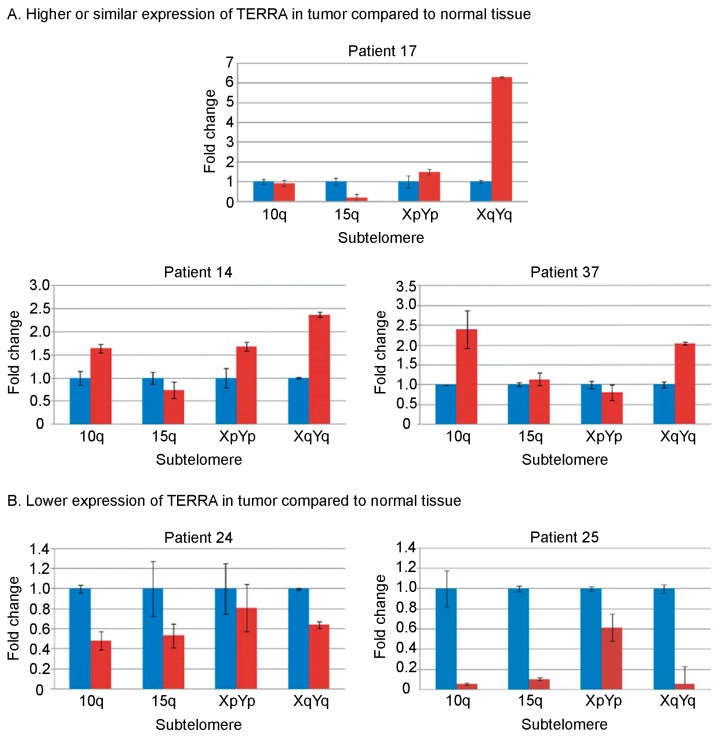
Telomere-specific TERRA levels by qRT-PCR (quantitative Real Time-Polymerase Chain Reaction) in 5 HNSCC patients. Quantitative RT-PCR experiments were carried out to measure of TERRA expression from 10q, 15q, XpYp and XqYq telomeres. Tumor samples (red bars) and samples from adjacent nontumoral tissue (blue bars). For each patient, TERRA expression level in the normal tissue is set to 1 and used as reference. For each sample, three replicates were amplified. (**A**) Patients with higher or similar TERRA levels in tumor compared to normal tissue; (**B**) Patients with lower TERRA levels in tumor compared to normal tissue.

For five patients, we also performed qRT-PCR (quantitative Real Time-Polymerase Chain Reaction) to measure TERRA levels from four different telomeres (10q, 15q, XpYp, XqYq) [2,4,34]. We were unable to carry out qRT-PCR experiments for all the patients because the amount of tissue in most samples was insufficient. The results of the qRT-PCRs confirmed the trend shown by the slot blot experiments (Figure 2). In agreement with previous observations [14,34,35], TERRA expression varies at different chromosome ends.

In Table 1, clinical data of the patients are reported: sex, age, site of the original tumor, tumor stage according to the pTNM (pathological tumor/node/matastasis) classification, tumor grading, status of each patient at data cut-off of 15 December 2017, and disease-free survival (DFS) (in months). In oropharyngeal tumors, an immunological test for human papilloma virus (HPV) was performed and showed to be negative in all analyzed patients. Patients are grouped in two classes depending on TERRA expression: (A) higher or similar in the tumor compared to normal tissue; (B) lower in the tumor compared to normal tissue. Patients are listed according to DFS. DFS was calculated from the date of sampling to either death, progression, occurrence of a second malignancy of the head and neck district or to the cut-off of 15 December 2017; according to this definition, patient n. 19 has not been censored at the time of development of a completely different second primary cancer (i.e., a Hepatocellular Carcinoma, HCC), while patient n. 35 has been censored when he developed a second primary tumor in the head and neck district.

Twelve of the fourteen patients in which TERRA was higher or similar in the tumor (Table 1A) compared to normal tissue were alive and disease-free. One of these patients (n. 35), although alive at follow-up, 69 months after surgery, underwent surgery for a second primary tongue tumor 33 months after the first surgery. 

Strikingly, seven out of the nine patients in which TERRA level in the tumor was lower than in the normal tissue (Table 1B) died of the disease within three years after surgery. All three patients previously operated for a distinct head and neck tumor belonged to this group; of them, two had died (n. 6 and 26) and one was alive at the date of the last follow-up. Only two of the nine patients in which TERRA levels in the tumor were lower than in the normal tissue were alive, although one of them (patient 19) developed a second primary tumor, an hepatocarcinoma.

**Figure 3:**
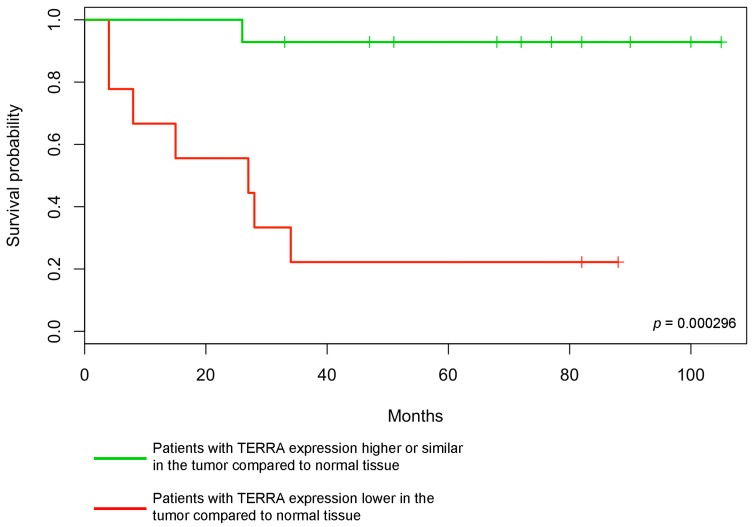
Disease free survival of 23 HNSCC patients. The probability of disease free survival, in months, in the two classes of patients is shown. Patients with TERRA expression higher or similar in the tumor compared to normal tissue (green curve), patients with TERRA expression lower in the tumor compared to normal tissue (red curve). The *p*-value of the log-rank test for the statistical significance of the difference between the two curves is shown.

In Figure 3, the Kaplan-Meier survival curve clearly shows that the disease-free survival is significantly lower in patients with low TERRA levels in the tumor (*p* = 0.000296).

## 3. Discussion

Little is known about the role of TERRA in human tumor development and progression. In two studies, lower levels of TERRA expression were observed in samples from different types of tumors compared to nontumor cells [3,13]. In particular, in astrocytoma, the extent of TERRA decrease was related to the progression [13], while in another study, elevated TERRA levels were observed in highly proliferating ovarian cancers [14]. In the same study, TERRA levels seemed to be higher in cancer cells than in adjacent non-tumor cells in a mouse medulloblastoma model. These observations suggest that telomere transcription is altered in cancer cells although it may play different roles in different tumor types. 

In the present work, we evaluated TERRA as a possible prognostic marker in HNSCC patients. To date, only clinical predictors of relapse have been identified for surgically resected HNSCC, irrespective of their site of origin, i.e., tumor dimensions, the presence of extracapsular spread in the case of locoregional lymph node involvement, and the presence of “close” surgical margins. The only biomarker of predictive and prognostic value identified so far for HNSCC is the presence of an oncogenic HPV infection in tumor cells, but its value is limited to HNSCC originating from the oropharyngeal region [36]. Indeed, in these tumors, the association of HPV positivity, T and N stage, and smoking habit (number of pack-years) contributes to stratify patients into three risk groups. Despite several efforts, no other biological or molecular marker has been validated to date [37].

We compared total TERRA levels, as measured by slot-blot hybridization, in HNSCC tumors with those in adjacent non-neoplastic tissue. To our knowledge, no data are available in which paired human tumors and adjacent normal tissues were compared. This type of comparison allows us to overcome the variability of TERRA expression among different individuals. In contrast to previous reports on other types of tumors, we did not observe a unique trend: TERRA expression levels in the tumor could be higher, similar or lower compared to the normal surrounding tissue. Although we tested only three lymph node metastases, total TERRA levels were always lower in the metastatic tissue compared to the normal tissue, independently of the TERRA level in the primary tumor.

Interestingly, patients in which TERRA levels were lower in the tumor had a worse clinical outcome than patients in the other class: seven out of nine died within 34 months postsurgery. Conversely, the majority of patients in which TERRA in the tumor was higher or similar compared to the normal tissue were alive and free of disease at the last follow-up: twelve out of fourteen were disease-free and one was alive but with recurrent disease. The striking difference in the clinical outcome of the two groups of patients is underlined by the low *p*-value of the Kaplan–Meier curves comparison (*p* = 0.000296). Furthermore, lower TERRA levels were found in tumor samples from the three patients where we analyzed a second HNSCC, which are commonly associated with a worse prognosis.

Besides analyzing total TERRA levels in all patients, we measured telomere-specific TERRA expression by qRT-PCR in a subset of patients. Although the general trend of expression mirrors the situation observed for total TERRA levels, single telomeres are expressed at different levels, supporting the idea that each chromosome end is regulated independently [34,35]. Our findings remark the need of specific studies aimed at determining the functional meaning of chromosome-specific telomere transcription and their relation to cancer progression.

A possible biological explanation of our findings is that TERRA dysfunction may be related to genomic instability, leading to the accumulation of oncogenic mutations, and/or to chromatin remodeling, affecting gene expression. 

Our two groups of patients are homogeneous in terms of age (median and range). It is well-known that telomere length decreases with age, however the correlation between telomere length and TERRA expression is controversial [34,35]. Due to the limited amount of tissue available, particularly for normal samples, we were unable to purify DNA and perform genomic analyses, including telomere length determination. Our results do not allow us to draw conclusions concerning a possible correlation between TERRA levels and telomere length in HNSCC patients. It will be important in the future to have access to large enough samples and to investigate these aspects. Since our results are based on the analysis of a relatively limited number of patients, to confirm and strengthen our findings, a larger number of HNSCC patients with different tumor stages have to be analyzed. Regarding oropharyngeal squamous cell carcinoma (SCC), comparing HPV-related and unrelated cases will help to clarify the role of tobacco- and HPV-mediated oncogenesis on telomere metabolism.

Finally, our results are based on the analysis of a single tumor type. Given the heterogeneous data published so far, TERRA expression and related parameters need to be evaluated in a large spectrum of cancer types to fully understand their involvement in the oncogenetic process.

In conclusion, the results of the present work suggest that TERRA levels can potentially represent a novel potential molecular prognostic marker for HNSCC, useful to develop therapeutic and follow-up strategies.

## 4. Materials and Methods

### 4.1. Patients Recruitment and Methods

Twenty-nine consecutive patients were enrolled in this prospective study, which was approved by the University of Pavia (22 April 2008) and by I.R.C.C.S. (Istituto di Ricovero e Cura a Carattere Scientifico) San Matteo University Hospital (23 April 2008 and 29 September 2010, Ethical Committee determination No. 2/D.G./1004, procedure No. P-20080012978). The main inclusion criterion of the study was squamous cell carcinoma of the head and neck (HNSCC) histology. In oropharyngeal cancers, HPV status was determined according to our validated algorithm [38]. Four patients were excluded from the present analysis due to the lack of follow-up data and two due to their non-HNSCC histology other than HNSCC (amenoblastoma of the jaw in one case and salivary gland adenocarcinoma in the other). Of the 23 evaluable patients, all but one, who presented with a liver metastasis from a SCC of the cervical esophagus, had a locally advanced disease. All the samples tested for TERRA expression derived from surgical removal of the primary tumor.

All patients received postoperative adjuvant therapy (either radiotherapy or combined chemo-radiotherapy), whenever necessary, according to current guidelines.

All patients signed an informed consent according to institutional requirements.

All the samples tested for TERRA expression were obtained at the time of the surgical removal of the tumor, and stored at −80 °C in RNA-Later Solution (Sigma-Aldrich, St. Louis, MO, USA) immediately after surgery. Samples of the tumor and of the adjacent normal mucosa were provided separately by the surgeons. In 3 cases, tumor samples from laterocervical lymph node metastases were also collected.

The survival rate was estimated using the Kaplan–Meier method. DFS was calculated from the date of sampling to either death, progression, occurrence of a second malignancy of the head and neck district or the date of the last available follow up (15 December 2017, if not indicated differently in Table 1). Survival was calculated as full months using the function DATEDIF (Start_Date, End_Date, m) in Microsoft Excel. The software R was used to draw the Kaplan–Meier curve for survival rate and to calculate the log-rank test for significance [39].

### 4.2. RNA Extraction and Purification

Tissue samples were placed in 1 mL of Qiazol reagent (Qiagen, Hilden, Germany) and mechanically homogenized using a Tissue Ruptor (Qiagen). Total RNA preparation was then carried out following the manufacturer’s instructions. RNA samples were treated twice with 1 U DNase I (Promega, Madison, WI, USA) per μg RNA at 37 °C for 30 min. Following each DNase treatment after, the RNA was purified with the RNA Clean and Concentration kit (Zymo Research, Irvine, CA, USA) according to manufacturer’s instruction.

### 4.3. Slot Blotting and Hybridization

All samples were diluted in three volumes of denaturation solution (26% formaldehyde, 7% of 20× SSC and 24% formamide) and incubated at 65 °C for 5 min, then, one volume of 20× SSC was added. For each patient, we blotted on the same filter (Amersham Hybond-N membrane, GE Healthcare, Little Chalfont, UK) RNA from the tumor and from the adjacent normal tissue, in at least three slots, with three RNA amounts ranging from 100 and 800 ng. To evaluate the presence of DNA contamination, the same amounts of RNA were also treated with RNase (RNase A, Sigma, over night at 37 °C) and blotted. This procedure did not allow us to compare TERRA levels in different tumors but only in the different tissues from the same patient. Total TERRA was detected using a ^32^P-α[dCTP]-labeled (CCCTAA)_5_ probe specific for the UUAGGG-containing strand. Hybridization was performed as previously described [2,4,34]. Radioactive signals were detected using a phosphorimager (Cyclone, Packard). When radioactive signal from the TERRA probe was decayed, filters were rehybridized with a probe for actin that was used as loading control. The ^32^P-α[dCTP]-labeled actin probe was obtained by PCR amplification of cDNA prepared from total RNA extracted from HeLa cells using the following primers: GGAAATCGTGCGTGACATTA and CTTCCTGTAACAACGCATCTC.

### 4.4. Data Analysis

TERRA and actin signals were quantified using the ImageJ software [40] according to standard procedures [41]. Signals produced by residual DNA contamination were evaluated quantifying the signals in RNase-treated samples and subtracting it from the value of the untreated sample. For each patient, TERRA signal in the normal tissue was used as reference and the signal in the tumor, and eventually in the metastasis sample, was normalized accordingly. After normalization with the actin signal, TERRA signals from the three amounts of RNA were used to calculate the average fold change value and the standard deviation. The significance of the fold change in the tumor relative to the normal tissue was evaluated using the two-tail Student’s *t*-test: the difference between TERRA expression in tumor tissue and healthy tissue was considered not significant when the *p*-value was higher than 0.05. Patients n. 14 and 3 were assigned to the first class (TERRA level similar in tumor and normal tissue) because the *p*-value was 0.0514 and 0.0653, respectively. Patients n. 34 was assigned to the second class because the *p*-value was 0.0366, therefore the difference between tumor and normal tissue was considered statistically significant. It must be pointed out that, since the p-values of these three patients were close to 0.05, their class assignment should be taken with caution because the experiments could not be repeated due to the limited amount of RNA available.

### 4.5. Reverse Transcription and qPCR

Real-time quantitative PCR experiments were performed as previously described [2,4,34]. For each sample, three replicates were amplified. Data were analyzed using the Opticon Monitor 3 software and Microsoft Excel. For each patient, the relative TERRA expression was calculated according to the ΔΔ*C*_t_ method [42] using the normal tissue as reference. 

Primer sequences used for qRT-PCR were:

- U6-F, GGAACTCGAGTTTGCGTGTCATCCTTGCGC;
- U6-R, GGAATCTAGAACATATACTAAAATTGGAAC;
- 10q-F, CCGTTTGCTGCCCTGAATAA;
- 10q-R, TCTGACGCTGCACTTGAACC;
- 15q-F, CAGCGAGATTCTCCCAAGCTAAG;
- 15q-R, AACCCTAACCACATGAGCAACG;
- XpYp-F, GCAAAGAGTGAAAGAACGAAGCTT;
- XpYp-R, CCCTCTGAAAGTGGACCAATCA;
- XqYq-F, CCCCTTGCCTTGGGAGAA;
- XqYq-R, GAAAGCAAAAGCCCCTCTGA.

## 5. Conclusions

We found a correlation between TERRA expression and tumor aggressiveness in HNSCC. In particular, patients with lower TERRA levels in the tumor compared to adjacent healthy tissue showed a worse clinical outcome than patients with similar or higher levels in the tumor. These results suggest that TERRA expression may be proposed as a novel molecular prognostic marker for HNSCC.

## Figures and Tables

**Table 1:** Clinical and histopathological data of the patients analyzed in this study. (A) Higher or similar expression of TERRA in tumor compared to normal tissue (*n* = 14); (B) Lower expression of TERRA in tumor compared to normal tissue (*n* = 9).

**No.**	**Sex/Age**	**Tumor Site**	**Staging (pTNM)**	**Grading**	**HPV Status**	**Date of Sampling**	**Status at 15 December 2017**	**DFS (Months)**
**A**								
9	F/71	Oropharynx	T4a, N0, M0	G2	Negative	15 April 2011	Dead of disease on 24 June 2013	26
35	M/67	Oropharynx	T3, N0, M0	G2	Negative	1 March 2012	Alive. On 15 December 2014, a second primary tumor (SCC of the tongue staged pT2, N2, M0, graded G2) was resected	33+
38	M/64	Tongue	T2, N0, M0	G2/G3	ND	16 December 2013	Alive, disease-free	48+
2	M/70	Hypopharynx	T4a, N2b, M0	G2	ND	10 September 2013	Alive, disease-free	51+
37	M/66	Oropharynx	T4a, N0, M0	G1/G2	Negative	26 March 2012	Alive, disease-free	68+
22	M/68	Tongue	T3, N0, M0	G2	ND	12 April 2010	Lost at follow-up on 9 May 2016, when was alive and disease-free	72+
28	F/79	Larynx	T3, N0, M0	G2/G3	ND	15 July 2011	Alive, disease-free	77+
20	M/59	Larynx	T4a, N0, M0	G2	ND	8 February 2011	Alive, disease-free	82+
7	M/55	Larynx	T4a, N0, M0	G2	ND	24 January 2011	Alive, disease-free	82+
18	M/79	Hypopharynx	T2, N2, M0	G2/G3	ND	17 January 2011	Alive, disease-free	82+
21	M/72	Tongue	T3, N1, M0	G2/G3	ND	1 June 2010	Alive, disease-free	90+
17	M/52	Oropharynx	T2, N0, M0	G2	Negative	24 May 2010	Alive, disease-free	90+
14	M/59	Larynx	T4a, N0, M0	G2	ND	20 July 2009	Alive, disease-free	100+
3	M/52	Pharynx	T4a, N0, M0	G3/G3	ND	26 February 2009	Alive, disease-free	105+
**B**								
**No.**	**Sex/Age**	**Tumor Site**	**Staging (pTNM)**	**Grading**	**HPV Status**	**Date of Sampling**	**Status at 15 December 2017**	**DFS (Months)**
10	M/65	Cervical esophagus	T2, N0, M1 (liver)	G2	ND	24 October 2013	Dead of disease on 21 March 2014	4
34	F/77	Hypopharynx	T3, N0, M0	G2	ND	26 November 2010	Dead of disease on 6April 2011	4
24	F/71	Oropharynx	T4a, N1, M0	G2	Negative	21 February 2011	Dead of disease on 19 November 2011	8
6	M/59	Hypopharynx ^1^	T4a, N0, M0	G2	ND	20 October 2011	Dead of disease on 10 February 2013	15
26	M/70	Oropharynx ^1^	T2, N0, M0	G2	Negative	7 July 2011	Dead of disease on 22 October 2013	27
11	M/58	Tongue	T3, N1, M0	G2	ND	28 September 2009	Dead of disease on 10 February 2012	28
27	F/63	Tongue	T3, N0, M0	G2	ND	11 July 2011	Dead of disease on 18 May 2014	34
19	M/70	Larynx	T4a, N2, M0	G2	ND	31 January 2011	Alive, disease-free (for head and neck tumor). On 6 March 2017 a second primary tumor (hepatocellular carcinoma, HCC) on a chronic HCV (hepatitis C virus) infection was diagnosed. The HCC was treated by means of RTFA and has not recurred to date.	82+
25	F/62	Larynx ^1^	T2, N2, M0	G2	ND	19 July 2011	Alive, disease-free	88+

^1^ All these patients were previously operated for a distinct tumor of the head and neck district; pTNM: pathological tumor node metastasis; HPV: human papilloma virus; DFS: disease-free survival; M: male; F: female; SSC: squamous cell carcinoma; HCC: hepatocellular carcinoma; ND: No Data.

## Abbreviations

Ct	Threshold cycle
DFS	Disease-free survival
HCC	Hepatocellular carcinoma
HCV	Hepatitis C virus
HNC	Head and neck cancer
HNSCC	Head and neck squamous cell carcinoma
HPV	Human papilloma virus
pTNM	Pathological tumor node metastasis
qRT-PCR	Real-time quantitative polymerase chain reaction
RTFA	Radio-frequency thermal ablation
TERRA	Telomeric repeat-containing RNA
